# Efficacy of Cal-Cemex as bone substitute for tibial plateau fractures

**DOI:** 10.1186/s13018-023-04323-1

**Published:** 2023-11-06

**Authors:** Andrea Pizzoli, Manuel Bondi, Laura Piotto, Nicola Tartaglia, Michele Saracino, Oleg Vyrva

**Affiliations:** 1 Department of Orthopaedics and Traumatology, ASST - Mantova, Carlo Poma Hospital, Strada Lago Paiolo 10, 46100 Mantua, Italy; 2 U.O.S.D. Traumatology, Hospital Miulli, Acqua Viva Delle Fonti, Bari, Italy; 3 Bone Tumor Department, Ukrainian National Academy of Medical Sciences, Sytenko Institute of Spine and Joint Pathology, Kharkiv, Ukraine

## Abstract

**Background:**

Various factors influence treatment and outcomes in tibial plateau fractures. Bone defects are among them. Many materials have been proposed to address this problem: allograft, bone–cements and various bone substitutes (BSM). Cal-Cemex (β-tricalciophosphate and polymethylmethacrylate) is a new hybrid bi-component BSM. A retrospective multicenter study was conducted based on the clinical experience of three European Hospitals, to demonstrate its clinical effectiveness, versatility and safety.

**Materials and Methods:**

From December 2016 to March 2022, 45 displaced tibial plateau fractures were treated with internal fixation and augmentation using Cal-Cemex. The average age was 55.9 years. According to Schatzker classification, we included 13 type II, 24 type III, 3 type V and 4 type VI fractures. The postoperative follow-up (FU) consisted of clinical and radiological examinations at 6 and 12 weeks and 1 year after surgery. A CT scan was performed preoperatively and 1 year after surgery. Full weight bearing was permitted after less than 6 weeks. Clinical data were collected from patient charts, while functional data were evaluated using the Rasmussen knee function score, the KOOS score and the Hospital for Special Surgery knee rating score (HSS), to evaluate the range of motion, axis and functionality of the knee.

**Results:**

The average FU was 42.8 months. CT scans taken at 1 year demonstrated a good surface osteointegration without radiolucent lines or osteolysis with good evidence of interdigitation and even bone ingrowth. At 1-year FU, the mean Rasmussen score was 24.7, the mean KOOS score was 90.7 and the mean HSS was 89.9 and the average full weight-bearing period 34.9. No patients had hardware failure or fracture secondary displacement.

**Discussion:**

Cal-Cemex combines biological features and good mechanical performances. It guarantees biocompatibility and osteoconductivity, although it is not fully reabsorbable; β-tricalciophosphate component gives macro- and microporosity that allow fluids to penetrate inside the material, to stimulate bone ingrowth.

**Conclusions:**

The study suggests that Cal-Cemex is an option for tibial plateau fractures, where augmentation and support are necessary for early full weight bearing. The absence of major complications, ease of application, the possibility to cut and perforate this material support its extensive use in bone augmentation for trauma cases.

## Introduction

Tibial plateau fractures present complex injuries involving knee articular surface and the meta-epiphyseal proximal tibial segment. The primary treatment objectives are to restore the mechanical and anatomical axis of the tibia and reduce articular fragments for the recover the range of motion and knee stability.

Various factors such as bone fragment displacement, bone defects, involvement of the subchondral bone and cartilage injury influence the treatment strategies and the outcomes [1–8]. Preoperative assessment with CT scan is crucial as it provides valuable information about the extent of the lesions and guides the optimal surgical approach for fracture reduction and stability [9, 10].

Open reduction and internal fixation (ORIF) using plates and screws is the preferred treatment for tibial plateau fractures. However, in complex fractures (Schatzker type V-VI) there are often challenges related to soft tissue conditions, especially in cases of high-energy trauma or elderly individuals, which affect the timing and the definitive surgical approaches.

Contemporary trends involve the use of low-profile plates and anatomic periarticular implants to perform ORIF technique [11–15]. Angular stability screws are utilized to achieve a better bone purchase, even in cases of osteoporotic bone. Whenever possible, minimally invasive techniques, such as MIPO (minimally invasive plate osteosynthesis), are employed to mitigate the risk of complications [13, 14]. The new Balloon tibioplasty technique was recently described, minimally invasive technique to reduce the fracture and restore the continuity of the articular surface [16].

External fixation serves as an option for complex fractures with soft tissue damage, providing temporary or definitive treatment and enabling a minimal invasive approach in order to reduce the incidence of major complication, such as deep infections [15, 17–19].

To ensure favorable outcomes, it is crucial to avoid articular surface incongruity, because it can lead to progressive cartilage degeneration, joint disfunction and early secondary osteoarthritis [20, 21].

For managing tibial plateau fractures, the evaluation of the bone defect is of the utmost importance.

In the past, autologous bone grafts harvested from the iliac crest were widely used to fill the void and support ORIF. However, this approach has associated morbidities, such as postoperative pain, hematoma and infection [22]. Additionally, a single donor site may be insufficient for cases with large defects requiring filling. Many authors recommend ARIF (arthroscopic reduction and internal fixation) for Schatzker type I, II, III and IV, while few papers report results about fractures type V and VI treated using ARIF [5, 6, 19, 23–26]. The main advantages of ARIF are low invasiveness and treatment of associated lesions, but the major risk remains iatrogenic compartment syndrome, in particular in those cases with high level of capsular lesions (type V and VI).

### Bone substitutes for bone void filling

Several materials have been proposed to fill subchondral voids and support articular reduction, including allograft (chips or struts), bone–cements and various bone substitutes material (BSM) [22, 27]. Calcium phosphate and calcium sulfate are commonly used due to their good biocompatibility and osteointegration properties. However, they have limited resistance to compressive stress as demonstrated in biomechanical tests [28–30] and clinical series [31–33]. Injectable paste forms of these materials are also available, allowing percutaneous application through a drill hole in the proximal tibial metaphysis for cases managed with percutaneous or indirect reduction. However, improper distribution and potential intra-articular leakage pose risks and may require revision operation primarily due to limited radiopacity [34, 35].

Due to its higher mechanical strength and elastic modulus, PMMA alone is not an optimal choice for repairing cancellous bone tissues, even if it remains one of the most used biomaterials in orthopedic and spinal surgery.

There are several composite bone–cement systems that can be developed with PMMA to reduce its complications and improve its biological properties for bone regeneration [36].

### Introducing a new bone substitute Cal-Cemex

In recent years, a new bone substitute with a hybrid formulation, β-tricalciophosphate (β-TCP) and polymethylmethacrylate (PMMA) has become commercially available. Cal-Cemex (Tecres S.p.A., Sommacampagna, VR, Italy) has been designed to serve as a filler for bony voids or defects and as a potential augmentation material for percutaneous screws or hardware. By using Cal-Cemex, meta-epiphyseal defects can be effectively filled, preventing residual metaphyseal gaps, thanks to its radiopacity as a bone void filler.

In our study, we have tried to demonstrate the clinical efficacy of Cal-Cemex by recording data relating to biocompatibility and osseointegration, as well as its ability to prevent joint failure or secondary re-dislocation in proximal tibial fractures after early weight bearing. We also tried to define possible complications or contraindications associated with its use.

## Material and methods

A retrospective multicenter study was conducted based on the experience of three European centers: Carlo Poma Hospital—(Mantua, Italy), F. Miulli Regional General Hospital (Bari, Italy) and Sytenko Institute (Kharkiv, Ukraine). The study protocol received approval from local ethics committees.

Between December 2016 and March 2022, 45 displaced tibial plateau fractures, in 44 patients underwent internal fixation and augmentation using Cal-Cemex, with one case involving bilateral fractures.

**Fig. 1:**
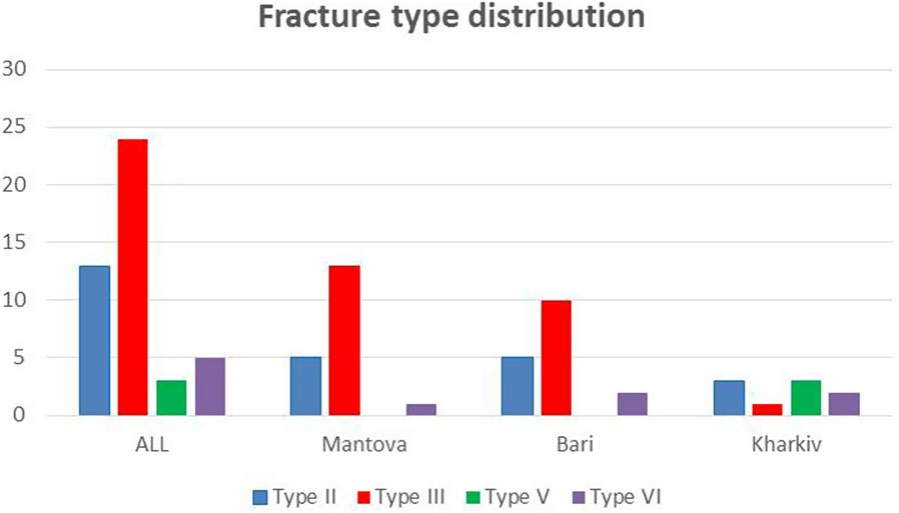
Fracture distribution according to Schatzker classification (all patients and per center)

The average age of the 44 patients was 55.9 years (range 26–82) at the time of surgery. Eighteen patients were male and 27 female: 14 right knee, 31 left knee **(**Table 1**)**. Preoperative immobilization was achieved with a posterior splint at emergency and accident department before admission at the hospital, and CT scans were obtained for fracture classification and surgical planning. All fractures were evaluated according to Schatzker classification: We included 13 type II, 24 type III, 3 type V and 5 type VI (Fig. 1).

**Table 1:** Patients’ data, fracture type, surgical treatment and outcomes scores

Pt	Initials	Sex	Side	Age	Fx type	Surgery	FWB (days)	KOOS	Rasmussen	HHS	FU (months)
1	V.P	M	L	59	III	LCP	40	91,1	21	85	51
2	A.T	M	L	55	VI	LCP	40	91,7	21	90	51
3	R.T	M	R	74	III	LCP	40	90,5	21	90	50
4	A.D	M	L	26	III	Screws	30	91,1	24	90	64
5	M.F.M	F	L	73	III	LCP	30	90,5	24	85	63
6	L.R	F	L	81	III	Screws	30	90	23	85	62
7	G.M	M	L	58	II	Screws	30	91,1	21	89	46
8	B.M	F	R	70	II	LCP	30	92,3	24	89	43
9	M.V	M	L	60	III	LCP	30	91,1	28	85	43
10	C.B	F	L	75	III	Screws	30	92,3	24	89	43
11	A.M.G	F	R	27	III	Screws	30	90	29	96	33
12	R.V	F	L	54	III	Screws	30	90	27	81	33
13	M.P	M	L	40	II	LCP	30	89	29	83	26
14	L.C	F	L	73	II	Screws	30	84	26	85	25
15	G.B	F	L	74	II	Screws	30	84	26	85	24
16	M.A.L	F	R	56	III	Screws	30	88	27	90	17
17	C.A	M	R	42	III	Screws	30	90	29	90	15
18	E.C	F	R	45	III	Screws	30	89	29	85	12
19	A.B	F	L	56	III	Screws	30	90,5	27	90	12
20	T.M	M	L	33	II	LCP	30	90	23	90	70
21	S.V	M	R	61	III	LCP	30	95	27	95	63
22	S.P	M	L	35	III	LCP	30	100	27	100	63
23	P.G	M	L	46	VI	LCP	30	100	27	100	70
24	L.M	F	R	76	III	LCP	40	100	27	95	55
25	M.A	F	L	42	II	Screws	30	90	27	90	55
26	D.A	M	R	62	III	LCP	30	95	27	90	54
27	P.L	M	L	45	II	LCP	30	95	21	95	53
28	R.I	F	R	82	III	LCP	30	90	18	90	53
29	G.A	F	L	69	III	LCP	30	90	21	90	52
30	T.L	F	L	53	II	Screws	30	95	23	90	48
31	M.F	M	L	52	III	LCP	30	90	23	95	48
32	I.G	F	L	60	III	LCP	30	90	23	95	48
33	I.G	F	L	60	III	LCP	40	90	18	90	48
34	L.V.M	F	R	55	III	Screws	40	90	21	90	48
35	D.F.G	F	L	48	VI	Screws	40	90	23	90	48
36	M.G	F	R	77	II	LCP	40	90	23	90	47
37	VS	M	L	33	II	LCP	30	90	28	90	37
38	AA	F	R	68	II	LCP	40	95	24	95	38
39	IV	F	L	55	II	LCP	30	100	27	100	22
40	TM	F	L	57	V	LCP	50	90	25	90	18
41	SA	F	L	54	V	LCP	60	70	20	80	37
42	IS	M	L	27	VI	LCP	60	65	24	70	24
43	NV	F	R	60	V	LCP	50	100	27	95	34
44	LV	F	L	74	III	LCP	30	90	27	95	64
45	VV	M	L	35	VI	LCP	60	95	27	95	16
				55,9			34,9	90,7	24,7	89,9	42,8

*LCP* locking compression plate; *CS* Cannulated screws; and *FWB* full weight bearing

The surgical procedures consisted in:

- 16 percutaneous reduction and screwing associated with ARIF and Cal-Cemex as void filler and augment;
- 29 open reductions associated with ORIF and Cal-Cemex as void filler and augment.

### Application technique

**Fig. 2:**
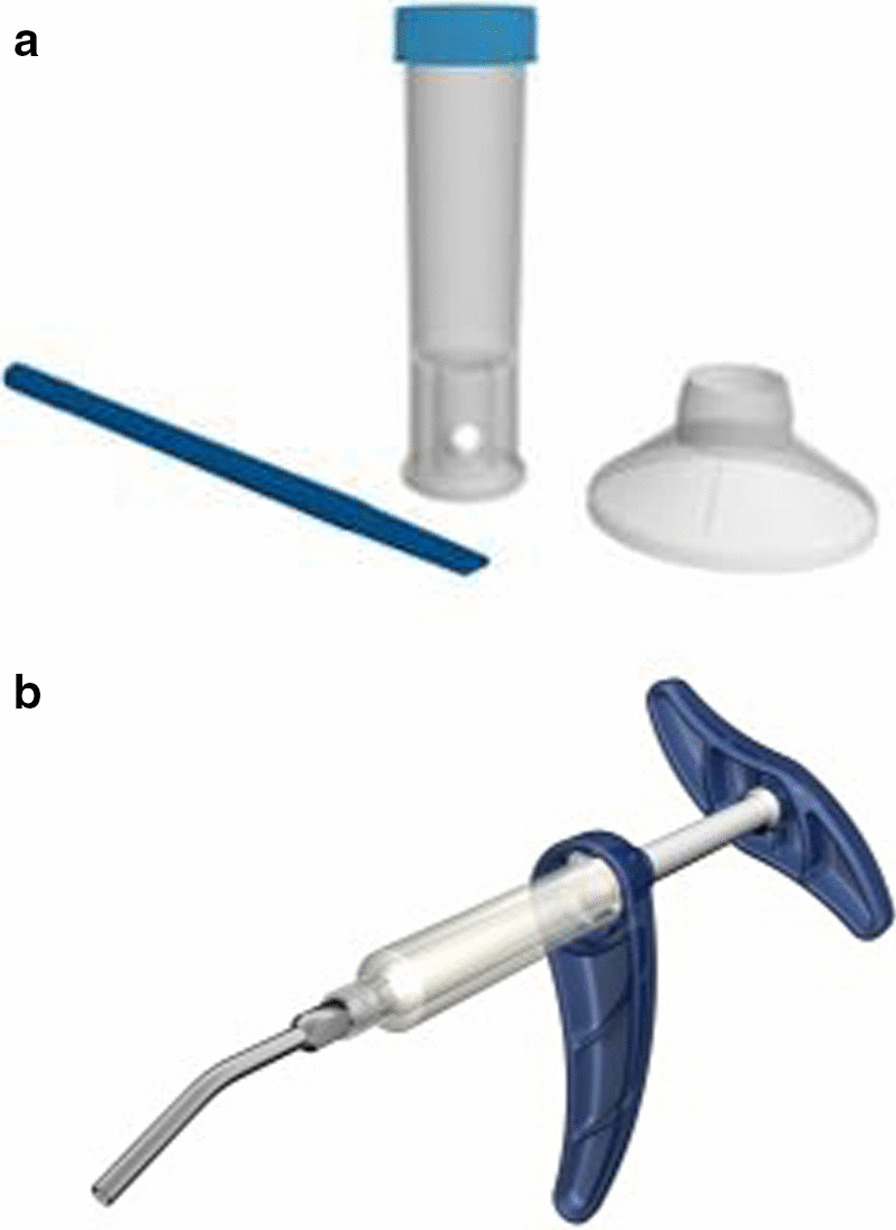
Shakit® (mixing device, **A**); Xtruder® (delivery device, **B**)

Cal-Cemex® is prepared from two separate sterile components (powder and liquid) using a simple mixing technique using a dedicated device (Shakit®) (Fig. 2A). The resulting homogenous paste is delivered through another dedicated device (Xtruder®) which includes a syringe and a cannula connected to a T-handle product pressurization (to facilitate handling) (Fig. 2B).

Tourniquet application and surgical field drying are essential before filling the dry cavity.

**Fig. 3:**
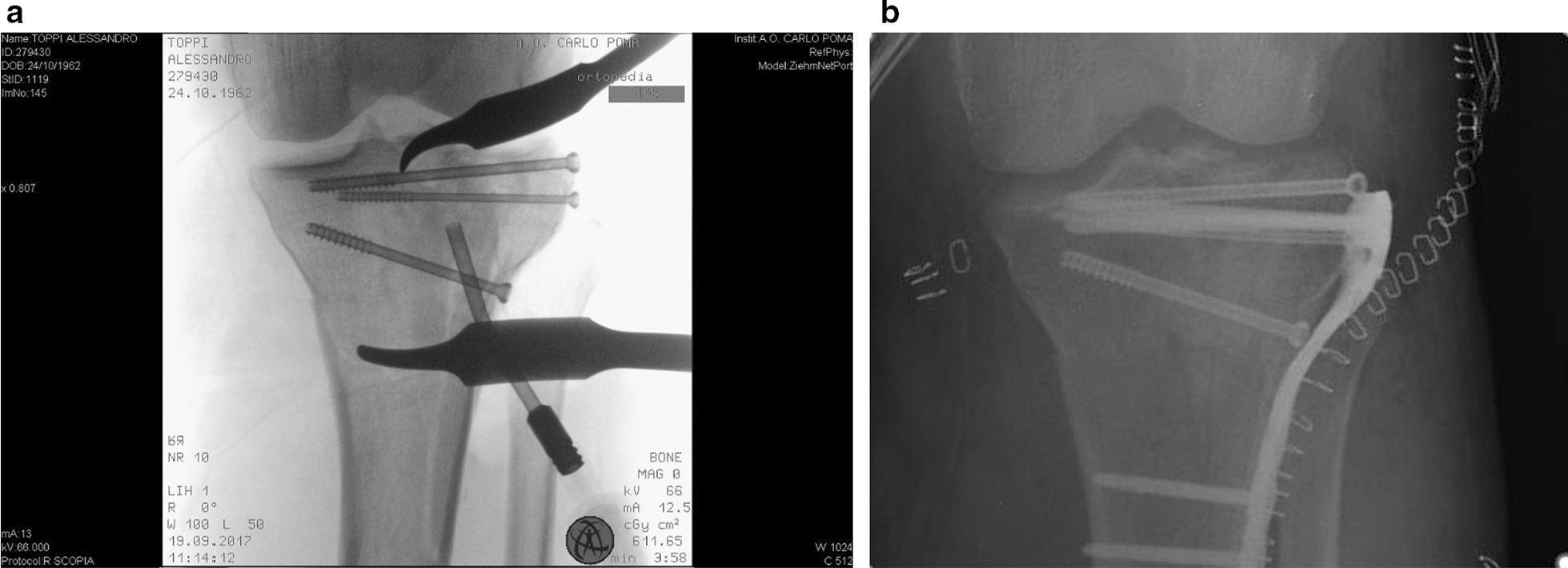
Injection is performed under fluoroscopic control to check volume distribution and avoid intra-articular leakage (**A**); after polymerization Cal-Cemex can be drilled (**B**)

Percutaneous injection is performed under fluoroscopic control to ensure optimal volume distribution and prevent intra-articular leakage (Fig. 3A). After polymerization, the paste solidifies into a cement-like substance that can be drilled or cut without compromising its biomechanical properties (Fig. 3B).

Digital pressurization during the application process facilitates interdigitation between Cal-Cemex, trabecular bone and hardware.

### Osteosynthesis description

Standard techniques were used to get open anatomical reduction of the articular surface in 20 cases, through an anterolateral + posteromedial approach. In 25 cases, indirect reduction was achieved by minimally invasive approaches using clamps and/or elevators.

After reduction (evaluated through a standard sub-meniscal exposure or under fluoroscopy and/or arthroscopy), k-wire was placed to secure the main fragments, keep the reduction and facilitate definitive fixation.

Buttress plate osteosynthesis using anatomic plates with locking and conventional (non-locking) screws was performed in 29 cases while internal fixation with AO cannulated cancellous screws (6, 5 or 7 mm) in 16 cases. The buttress plate was used in cases where there was major fracture comminution or complete displacement of the medial or lateral tibial plateau metaphyseal. In our cohort, we have used Cal-Cemex as void filler of bone defects after definitive osteosynthesis to obtain metaphyseal support in 16 cases, while in 29 cases it was applied percutaneously, after temporary fixation with k-wire, to obtain cannulated screw augmentation (considering that it can be drilled).

No patients underwent arthroscopy postoperatively to evaluate for cement extravasation.

### Follow-up

Passive joint motion was initiated one week after surgery with physiotherapist-assisted exercises, and full weight bearing was permitted within an average of 6 weeks (range 30–60 days) with a few exceptions (type V and VI fractures) supported by an articulated brace (Table 1). Postoperative follow-up consisted of clinical and radiological examinations at 6 weeks, 12 weeks and 1 year after surgery. Some patients underwent a CT scan at the 1-year mark to assess fracture reduction, healing, interdigitation and osteointegration. Clinical data were collected from patient charts, while functional data were evaluated using the Rasmussen knee function score [37], the KOOS score [38] and the Hospital for Special Surgery knee rating score (HSS) [39] comparing the injured side with contralateral side at any follow-up. All this is to evaluate the range of motion, the axis and the pain for every patient.

## Results

The average follow-up was 42,8 months (range 3–70). Early weight-bearing time was allowed on average 3–5 days post-surgery, while full weight bearing was allowed after an average period of 34.9 days (range 30–60). At 1-year follow-up, the mean Rasmussen knee function score was 24.7 (range 18–29), the mean KOOS score was 90.7 (range 65–100) and the mean HSS was 89.9 (range 70–100) (Table 1). The measurements demonstrated progressive improvement during subsequent follow-up visits (6, 12, 52 weeks). None of the patients experienced major complications such as cement leakage, complex regional pain syndrome, neuroma or exostosis formation, not deep infection or local inflammatory reactions. Soft tissue complications such as wound infection and wound dehiscence were also not registered. There were no cases requiring reoperation or implant removal for any reason.

**Fig. 4:**
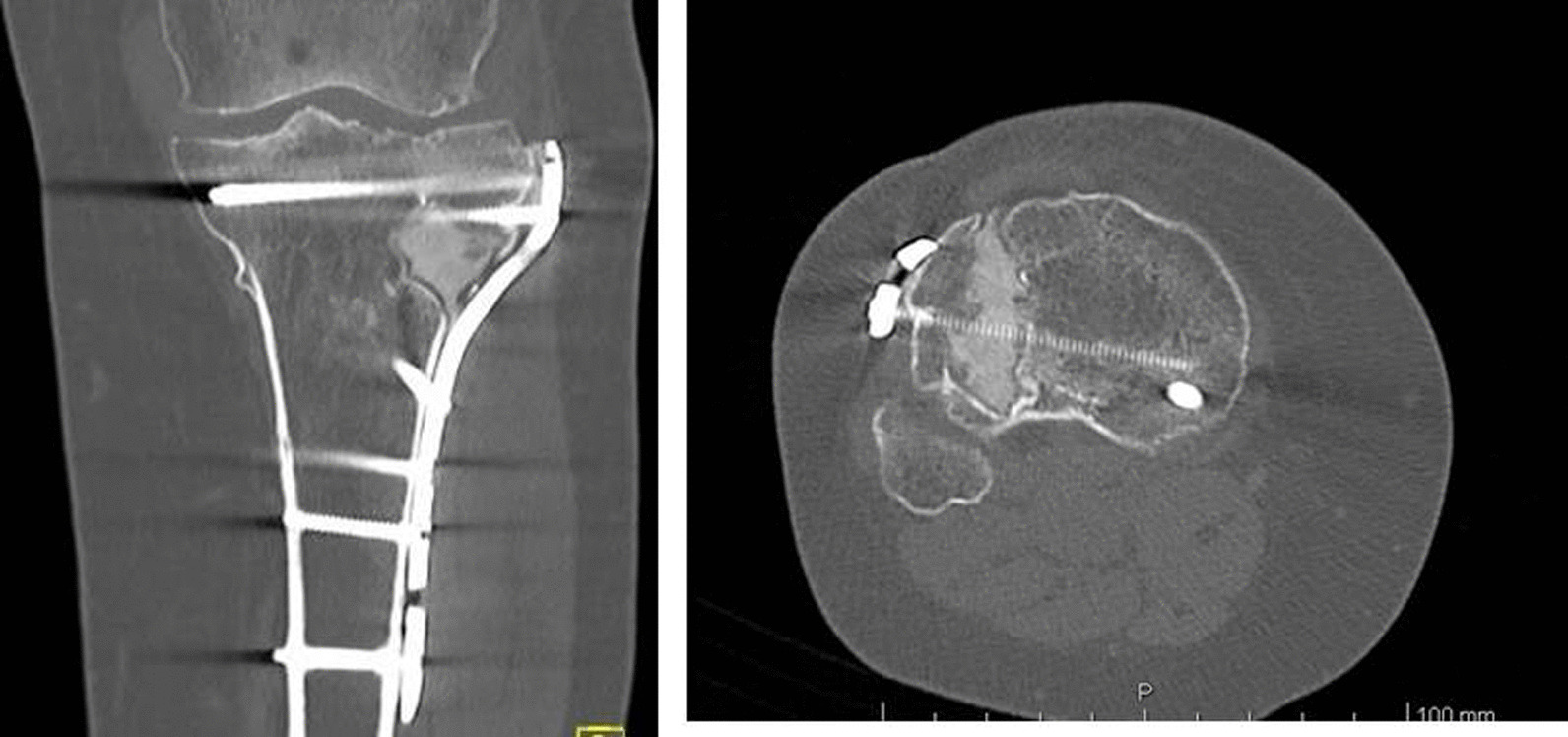
One-year postoperative CT scan demonstrates a partial surface integration without radiolucent lines or osteolysis with good evidence of interdigitation and even osteointegration

At the 1-year follow-up, no patients exhibited screw mobilization or fracture reduction loss. CT scans taken at this time point revealed good surface osteointegration without radiolucent lines or osteolysis, providing evidence of interdigitation and bone ingrowth (Fig. 4).

### Cal-Cemex description

#### Microscopic structure and porosity

Cal-Cemex exhibits a unique microscopic structure with interconnected macropores and micropores, filled with β-TCP and linked by microcanals, supported by the spongy matrix of PMMA [40]. This distinctive porous surface features a stringy appearance, numerous microreliefs, cavities and pores.

The micropores have an average diameter ranging from 10 to 15 μm, while macropores range from 100 to 250 μm. These dimensions facilitate primary interdigitation and improve secondary bone ingrowth, closely resembling cancellous bone trabeculae (ranging between 200 and 300 μm).

#### Biological integration and biomechanical advantages

The resorption of β-TCP and resulting lacunae promote the integration of Cal-Cemex with the native trabecular structure, enabling cellular colonization and faster bone formation [40]. Preclinical studies provide evidence of the material positive biocompatibility and osteointegration properties. Biomechanical tests demonstrate that Cal-Cemex outperforms traditional β-tricalcium phosphate due to the improved mechanical performances resulting from the PMMA component. For instance, its static mechanical compression strength is 50 MPa compared to 100 MPa of traditional PMMA, while bending strength and bending modulus are 30 MPa versus 60 MPa and 1000 MPa vs 3000 MPa, respectively.

Despite the lack of clinical data to support the surgeons to use this product, biomechanical tests confirm the indication to use Cal-Cemex as bone void filler and/or augment of osteosynthesis.

## Discussion

Management of meta-epiphyseal defects in tibial plateau fractures remains a subject of debate, considering factors such as the type of lesion, the extent of bone loss and the quality of patient’s bone tissue [40, 41].

### Autologous bone grafting

For many years, autologous iliac bone grafts have been considered the gold standard for managing metaphyseal bone defects associated with intra-articular fractures. These grafts offer advantages such as availability, cost-effectiveness, structural support and osteoinductive biological effectiveness. However, the use of bone grafts requires additional surgical procedures and may lead to complications at the donor site including pain, avulsion fractures of the anterior superior iliac spine (ASIS), hematoma, iatrogenic infection, herniation, gait disturbance, cosmetic deformity, sacroiliac joint instability and ureteral injury. Harvesting from the anterior iliac bone carries the risk of injury to the lateral femoral cutaneous nerve and the superior crural nerves [42–46]. Several studies have reported complications and functional impairments associated with autologous iliac bone grafts, further contributing to a decline in their use, particularly with the introduction of angular stable anatomical plates that offer superior mechanical performances even in cases of poor bone quality.

Medina et al. [47] reported avulsion fracture of the ASIS. Goulet et al. [48] reported that 38% of their 87 patients had pain at 6 months after the harvest of autologous iliac bone. Silber et al. [49], in a study of 134 patients, found that the rate of functional impairment ranged from 7% (for household chores) to 13% (for walking) at an average of 4 years after autologous iliac bone grafting. In another study of bone grafting of humeral shaft non-unions, Hierholzer et al. [50] reported complications in twenty out of forty-five patients who had autologous bone grafts (44%).

For this reason and for the introduction in the market of the angular stable anatomical plates, the use of autologous bone graft has dropped in the last decade, while bone substitutes applications have increased and overcome procedures based on allograft or autograft [51].

### Bone substitutes

Allografts, on the other hand, exhibit slow and incomplete osteointegration [52], while cancellous grafts and/or hydroxyapatite blocks or granules allow full weight bearing after an extended postoperative period [41, 53, 54].

Calcium phosphate or sulfate cements are reliable alternatives due to their ease of use, biocompatibility and osteoconductive properties, although they suffer from poor mechanical performances, such as low compression strength [40, 55, 56].

Among biological cements described in the literature [51–56], Cal-Cemex® (Tecres, Verona, Italy) stands out as a hybrid bone substitute that combines the biological features of beta-tricalcium phosphate (β-TCP) with the superior mechanical performance of polymethylmethacrylate (PMMA). Cal-Cemex combines the mechanical strength of PMMA and the biological features of beta-TCP: The mechanical performances are achieved immediately after polymerization and do not change over time [40], and the biological performances improve over time: The beta-TCP microgranules create a structure of interconnected pores which guarantee a diffuse microporosity responsible for the capillarity of the material, while the beta-TCP macropores promote osteoconduction and bone ingrowth over time through interconnected pores [57]. This microporosity enables interdigitation with bone, while macroporosity facilitates osteointegration. Another advantage of Cal-Cemex is its radiopacity, which aids in optimizing percutaneous filling, preventing articular leakage and facilitating post-op X-ray imaging. Eventually, Cal-Cemex can be easily perforated and/or cut after polymerization, making it convenient for hardware application or future arthroplasty procedures. From a clinical perspective, the multiplanar mechanical strength of the PMMA component allows for early weight-bearing times, rapid functional recovery and a swift return to daily activities without the risk of secondary fracture displacement.

### Results obtained with Cal-Cemex

In our multicenter experience, patients treated with Cal-Cemex achieved full weight-bearing recovery within an average of 34.9 days, with the longest recovery period observed in cases involving Schatzker V and VI fractures (Table 1). With other bone grafts, the average weight-bearing time reported in the literature varies from 6 to 12 weeks [28–31].

The clinical scores obtained in this study were like those reported in other studies on proximal tibial fractures [19, 23, 28, 35]. The worse scores reported (KOOS under 70 and Rasmussen under 20) are relative to type V or VI fractures (cases 41, 42) or to elderly patients with associated arthritis (case 28) or to polytrauma patient (case 33). In case 5, we had disagreement between clinical results and scores related to polymyalgia and depression.

In a recently published preclinical study on pigs, the authors demonstrated that Cal-Cemex samples implanted in the distal epiphysis of the femur and removed after one year had assumed macroscopically the same color of the surrounding cancellous bone as a consequence of osteointegration. Moreover, the specimens did not show any solution of continuity or irregularity as demonstration of optimal mechanical resistance to weight bearing. (Pigs increased their weight from 60 to 220 kg over the year of implantation of the samples.) Microscopically, the absence of inflammatory and necrotic aspects in the tissues surrounding the injected cement support its biocompatibility and the absence of thermal effects due to polymerization [40].

Evidence of osteointegration, lack of inflammatory and necrotic aspects of the surrounding tissues and absence of thermal effects were confirmed by our clinical study confirming the evidence of the previous preclinical studies [40, 57].

**Fig. 5:**
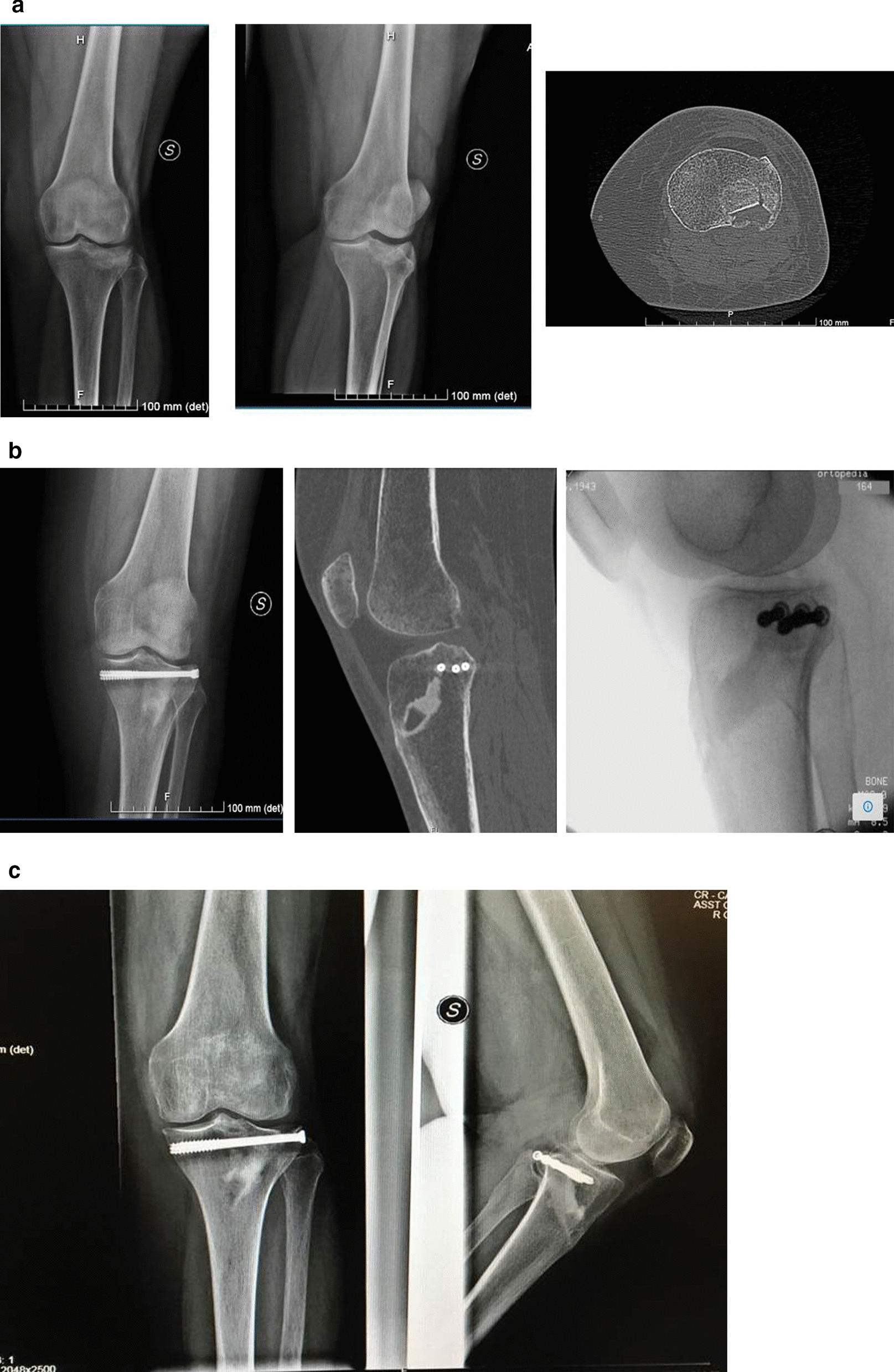

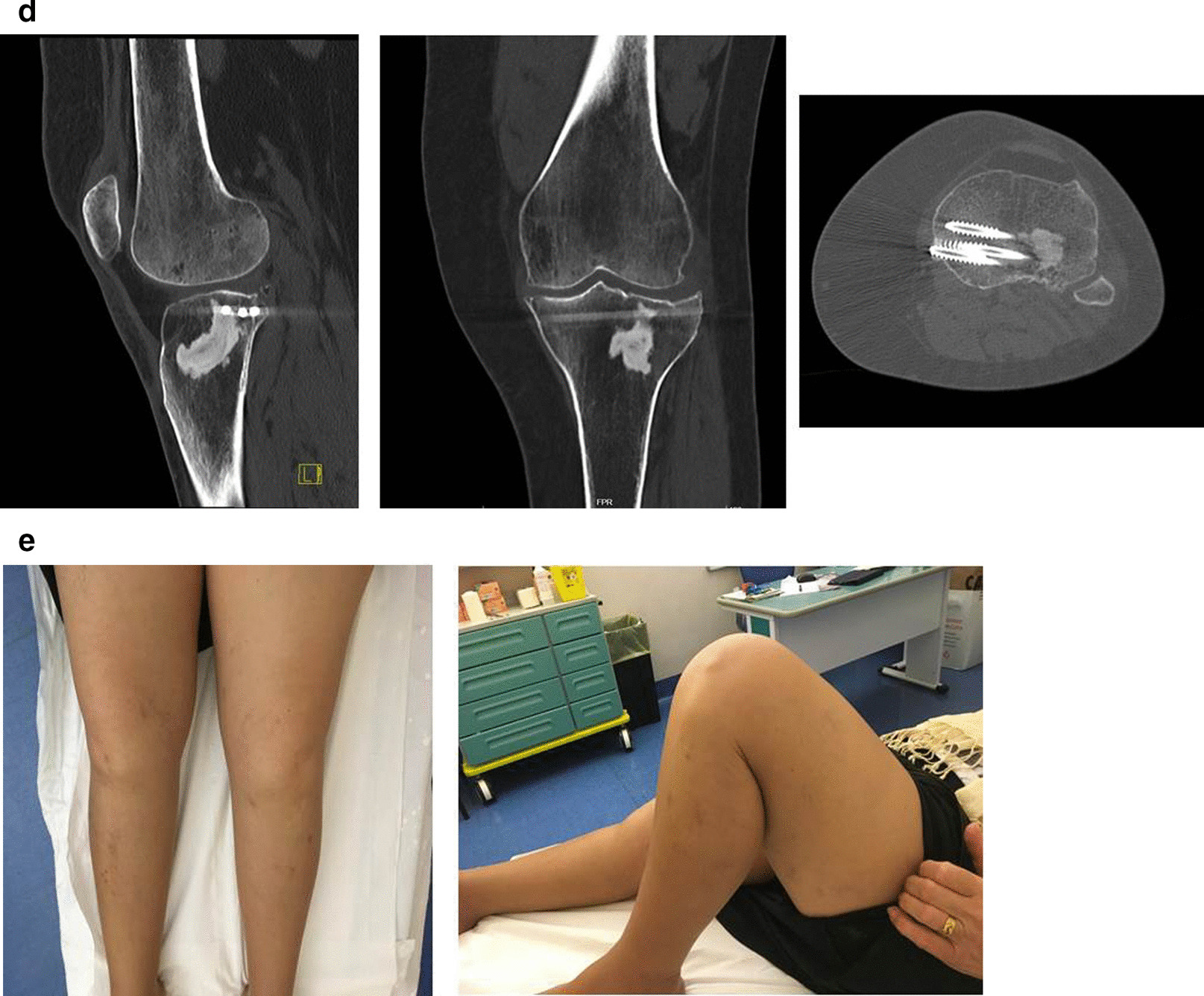
A 73-year-old woman. **A** Preoperative X-ray and CT scan demonstrating Schatzker type III fracture. **B** Postoperative X-ray: anatomic reduction of articular surface and Cal-Cemex. **C** Six-month postoperative X-ray: no significant change in bone–cement interface or loss of reduction, good bone–cement interdigitation. **D** Six-year postoperative CT scan demonstrating osteointegration and no loss of reduction. **E** Six-year clinical evaluation: good axis and range of motion

CT scans performed in some patients at 1-year follow-up (Fig. 4) did not show any evidence of radiolucency or osteolysis that would separate the Cal-Cemex from the bone. This is even more evident in the patient of Fig. 5 where CT scan was taken 6 years after surgery (Fig. 5A–E).

The complication rate for tibial plateau fractures ranges between 2 and 23.6% [58–61]. Many studies have shown a reoperation rate for any reasons of 0 to 20% [58–60, 62] with more frequent problems related to infection, malalignment, arthrofibrosis and a nonunion rate ranging from 0 to 10% [58, 60, 62]. In our study, we did not encounter any of these complications. Notably, no major complications such as infection, inflammation, necrosis, fibrosis or foreign body reaction were encountered, and no patient required postoperative arthroscopy due to cement leakage or subsequent arthroplasty procedure.

The strength of this study lies in its multicenter design and long follow-up period. However, limitations include its retrospective nature, small sample size and inclusion of different types of lesions; this did not allow a case selection and a linear comparison of the same cases type. Further studies are needed to demonstrate the clinical superiority of Cal-Cemex compared to other bone substitutes. While current BSM such as bioglass, β-TCP and hydroxyapatite offers good biological properties, their biomechanical performances remain limited. While they serve as fillers with favorable osteoconductive properties, their suitability for screw augmentation and metaphyseal support is compromised. Ideally, a bone substitute should have mechanical properties closer to natural bone, enable full integration, undergo reabsorption and potentially serve as a delivery system for factors that stimulates bone healing or for antibiotics that prevent or treat infections.

The findings of this study confirm the safety and effectiveness of Cal-Cemex in the treatment of traumatic meta-epiphyseal bone losses. However, further studies with long follow-ups will be necessary to test the effectiveness of this BSM also in other anatomical areas, even to confirm the advantages of early joint recovery and a reduction in no weight-bearing times for the lower limbs, as well as to demonstrate its usefulness in situations such as neoplastic bone loss and replacement surgery.

## Conclusions

The use of bone substitutes to compensate for bone loss both in trauma and orthopedic cases is still increasing.

Cal-Cemex (β-TCP + PMMA), a relatively new bone substitute, combines biological features and good mechanical performances (strength and elastic modulus of PMMA), making it an attractive option, particularly for treating unstable fractures with bone loss and/or poor bone, such as tibial plateau fractures.

Cal-Cemex showed biocompatibility, osteoconductivity and osteointegration, although it is not fully reabsorbable. Its mechanical performance, well-suited for augmentation and metaphyseal support, and its percutaneous application further contribute to its appeal.

The results of this retrospective multicenter clinical study suggest that Cal-Cemex is a suitable option for tibial plateau fractures, where augmentation and support are necessary for early full weight bearing, without the risk of any secondary fracture displacement.

Furthermore, the absence of major complications observer in our study, ease of application, the possibility to cut and perforate this material in case of hardware removal or future arthroplasty and the favorable medium-term clinical and radiological outcomes, although the study is retrospective, support its extensive use in bone augmentation and void filling, particularly for trauma cases. Further applications could include neoplastic bone loss and replacement surgery [63].

Further studies will need to be carried out to verify its usefulness and reliability even in the non-traumatological field.

## Data Availability

All data generated or analyzed during this study are included in this published article.
